# Prednisone reduction for metastatic castration-resistant prostate cancer with recurrent pulmonary tuberculosis: Case report

**DOI:** 10.1097/MD.0000000000025584

**Published:** 2021-04-16

**Authors:** Xiaojuan Zhu, Zhenguo Shi, Shegan Gao, Xiaohong Wang, Pei Wang, Chongzhao Kang, Fangzong Zhao, Peng Hou

**Affiliations:** Department of Radiation Oncology, The First Affiliated Hospital of Henan University of Science and Technology, Luoyang, China.

**Keywords:** advanced prostate cancer, prednisone, pulmonary tuberculosis, recurrence

## Abstract

**Introduction:**

Prednisone (10 mg/d) is often used in combination with docetaxel or abiraterone in the treatment of advanced prostate cancer. LATITUDE studies have confirmed that the combination of abiraterone and prednisone (5 mg/d) can be used for the treatment of newly diagnosed high-risk metastatic castration-sensitive prostate cancer, and have achieved satisfactory results. However, it has not been reported that abiraterone combined with prednisone (5 mg/d) in the treatment of metastatic castration-resistant prostate cancer (mCRPC).

**Patient concerns:**

Here, we present a case of high-risk advanced prostate cancer with old pulmonary tuberculosis (PTB). The patient developed a relapse of old tuberculosis in both lungs that were discovered following 14 months of continuous application of prednisone (10 mg/d).

**Diagnosis:**

The histopathological findings showed prostate adenocarcinoma carcinoma with a Gleason score of 10 (5+5). Further laboratory investigations were suggestive of positive mycobacterium tuberculosis complex DNA in pleural effusion and sputum.

**Interventions:**

The patient underwent endocrine therapy, chemotherapy of docetaxel plus prednisone, radiotherapy, and abiraterone combined with prednisone treatment, but he eventually developed into the mCRPC stage. Then, prednisone was reduced to 5 mg/d plus abiraterone, and combined with anti-tuberculosis treatment according to multi-disciplinary diagnosis and treatment.

**Outcome:**

Two months later, pleural effusion and atelectasis were relieved, and PSA was remained stable at a low level. The patient achieved complete remission.

**Conclusion:**

We cannot, with complete certainty, say that this patient, or any patient, developed old PTB recurrence due to the use of prednisone. Based on the current evidence, endocrine therapy is the foundation, radiotherapy can reduce the tumor load, and early application of abiraterone is beneficial to survival for the high-risk mCRPC. The long-term use of prednisone can be appropriately reduced in mCRPC with old PTB, and a satisfactory curative effect can be achieved. More prospective trials are warranted before a definite recommendation could be drawn.

## 1 Introduction

Prostate cancer is the most common malignant tumor of the genitourinary system in elderly men.^[1]^ In China, the incidence rate of prostate cancer is increasing year by year, and most patients are in the late stage of initial diagnosis, and the overall prognosis is poor.^[2]^ The research of different treatment options for advanced prostate cancer (APC) has become a hot issue at present. However, there are few studies on the dosage and safety of prednisone for APC. Prednisone is an essential drug for patients with APC and is often combined with new endocrine drugs (abiraterone) or adjuvant chemotherapy (docetaxel). Accordingly, the long-term use of prednisone will inevitably lead to glucocorticoid-related adverse events. Here, we report a case of old pulmonary tuberculosis (PTB) recurrence after comprehensive treatment of APC. The patient achieved complete remission after appropriate adjustment of drug dosage according to multi-disciplinary diagnosis and treatment (MDT). The therapeutic effect and safety of prednisone dose-related were reviewed, and the reference was hoped to be provided for clinical practice.

## 2 Ethics statement

Ethics statement is not applicable for case reports according to the Ethics Committee of the Henan University of Science and Technology Affiliated First Hospital, but informed consent was obtained from the patient for publication of this case report and the accompanying images.

## 3 Case presentation

**Figure 1:**
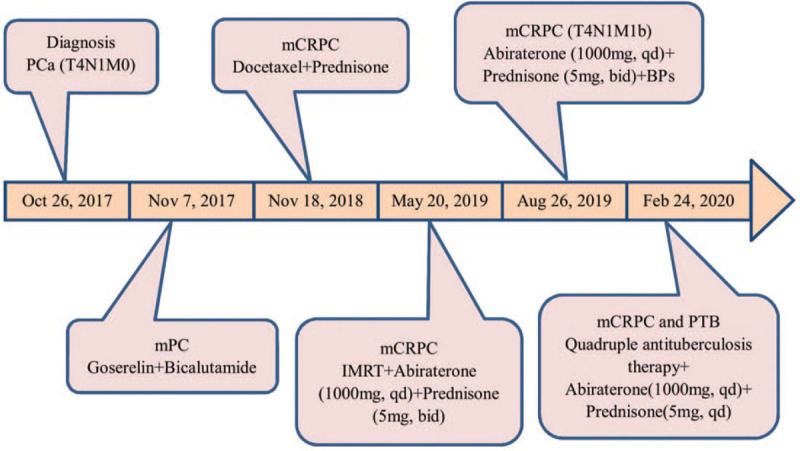
Timeline, diagnosis and treatment of patient at different time. BPs = bisphosphonates, IMRT = intensity-modulated radiotherapy, mCRPC = metastatic castration-resistant prostate cancer, mPC = metastatic prostate carcinoma, PCa = prostate cancer, PTB = pulmonary tuberculosis.

**Figure 2:**
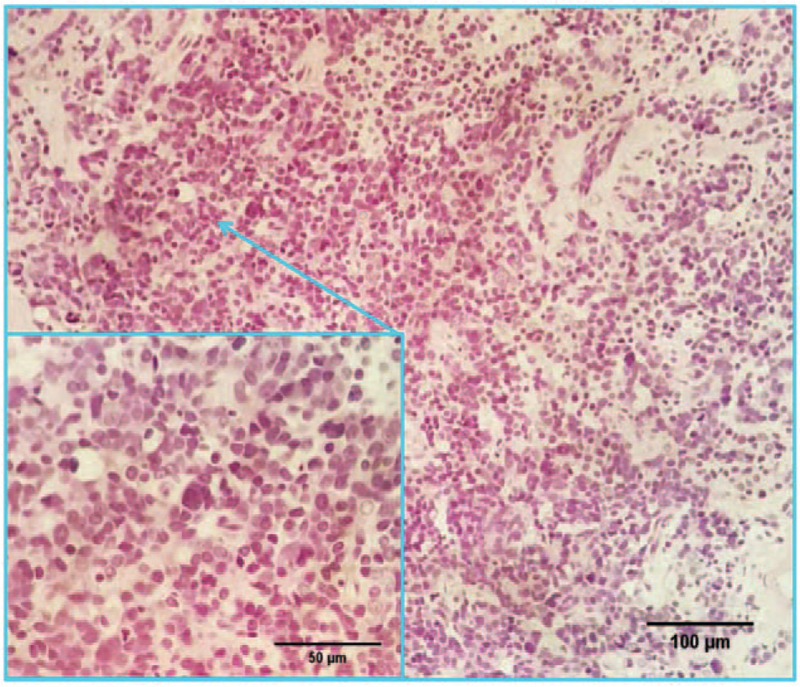
Histopathological findings showed a high cellularity tumor and prostate adenocarcinoma carcinoma with a Gleason score of 10 (5 + 5) (original magnifications ×200 and ×400). The resected tissue showed that the tumor cell nuclei are deep stained and characterized by heteromorphism and caryocinesia. HE = hematoxylin and eosin.

**Figure 3:**
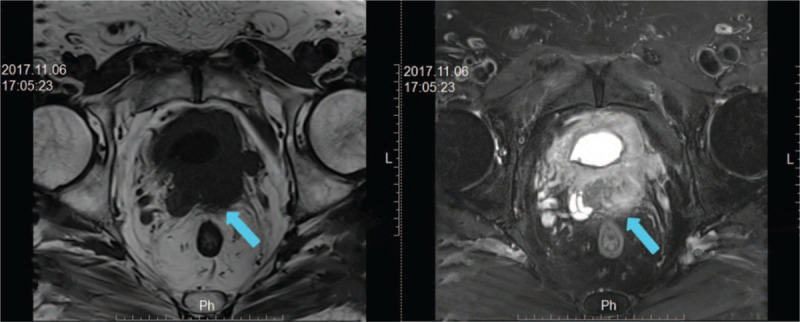
Magnetic Resonance Imaging (MRI) of the prostate revealed metastatic disease, the unclear capsule of the prostate, bilateral seminal vesicle invasion, rectal involvement, and multiple pelvic lymphadenopathies after transurethral prostatectomy and before medical treatment.

A 77-year-old male patient was admitted to the hospital with progressive dysuria for half a year and aggravating for 10 days on October 26, 2017 (Fig. 1). The history of old PTB was more than 40 years. Abdominal physical examination showed that the suprapubic bladder area was elevated. The digital rectal examination found that the prostate III degrees increased, the central sulcus disappeared, and the bilateral lobes were slightly hard. The color doppler ultrasound with which the patient presented indicated enlargement of the prostate and urinary retention, residual urine volume was 500 ml. Further laboratory investigation total prostate-specific antigen (tPSA) was 9.02 ng/ml, free prostate-specific antigen (fPSA) was 2.96 ng/ml, and fPSA/tPSA was 0.33. DR revealed old lung lesions and old PTB were more likely. The patient underwent electro-prostatectomy, and post-operative histopathological findings showed prostate adenocarcinoma carcinoma with a Gleason score of 10 (5 + 5) (November 07, 2017) (Fig. 2). Magnetic resonance imaging of the prostate showed metastatic disease, the unclear capsule of the prostate, bilateral seminal vesicle invasion, rectal involvement, and multiple pelvic lymphadenopathies (Fig. 3). Furthermore, the bone scan was normal. The final diagnosis was metastatic prostate carcinoma with a clinical-stage of T4N1M0 and old PTB.

**Figure 4:**
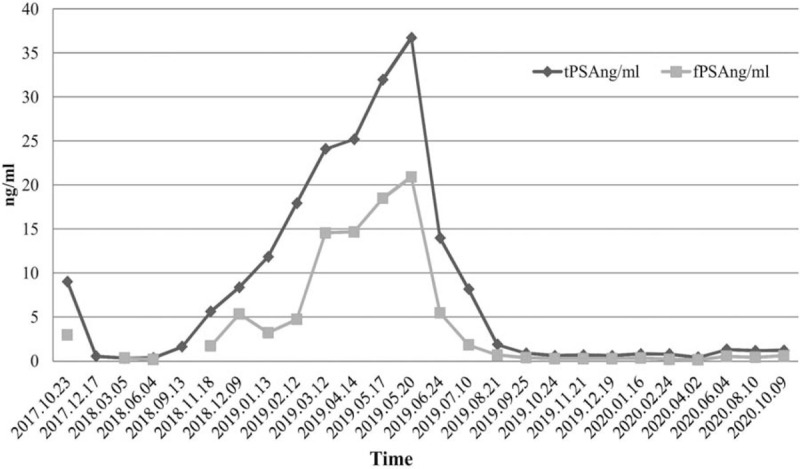
Timeline, the change of PSA level after various treatments.

Initially, goserelin plus bicalutamide as a first-line endocrine therapy program had been provided on November 7, 2017. After that, tPSA and fPSA showed a downward trend in more than 10 months (Fig. 4). The reexamination results of tPSA and fPSA were 5.63 ng/ml and 1.71 ng/ml respectively, which were considered as biochemical recurrence on November 18, 2018. As such, the patient chose docetaxel plus prednisone as second-line chemotherapy on December 9, 2018.

**Figure 5:**
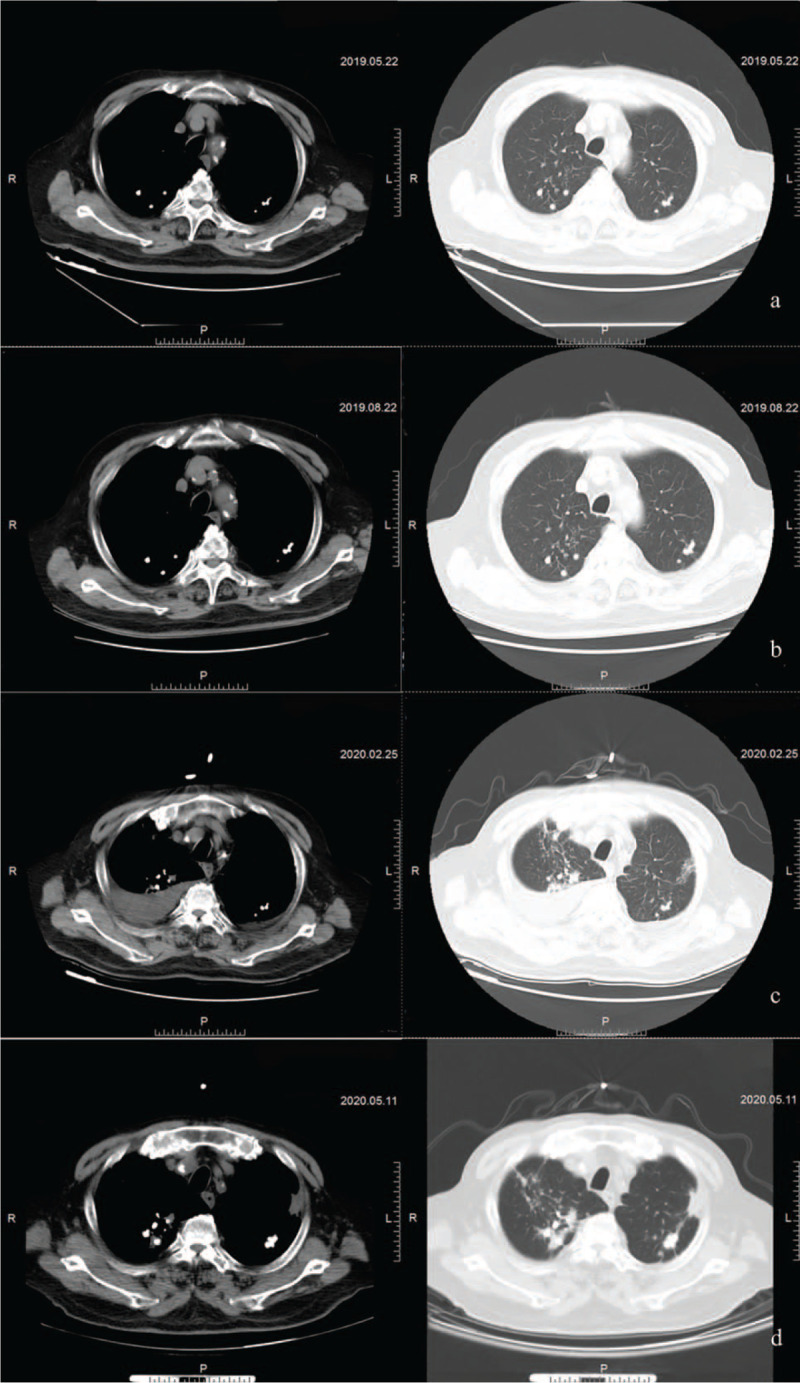
Chest computed tomography (CT) during treatment. (A) After 5 courses of docetaxel plus prednisone chemotherapy, the chest CT indicated that mild interstitial changes with a small amount of exudation, fibrous streak shadow, and signs of old pulmonary tuberculosis in both lungs. (B) After 3 courses of abiraterone plus prednisone, the chest CT displayed both lungs were the same as before. (C) Recurrence of tuberculosis after nine courses of abiraterone plus prednisone, the chest CT exhibited bilateral pleural effusion with atelectasis on February 25, 2020. (D) After 2 courses of anti- tuberculosis (TB) combine with abiraterone plus prednisone (5 mg/d), the pleural effusion were completely absorbed in both lungs.

After 5 chemotherapy courses, the PSA did not decrease, but it showed a continuous upward trend (Fig. 4). Bone scan showed no bone metastasis on March 15, 2019. However, tPSA was 36.75 ng/ml and fPSA was 20.92 ng/ml on May 20, 2019. Chest computed tomography (CT) showed that mild interstitial changes with a small amount of exudation, fibrous streak shadow, and signs of old PTB in both lungs (Fig. 5A). Thereafter, the patient underwent radical radiotherapy of prostate carcinoma, abiraterone (1000 mg, qd) combined with prednisone (5 mg, bid) treatment according to the discussion of MDT. Radiation therapy was delivered via high-energy (≥6 MV) linear accelerators, and a total radiation dose of 70Gy/35 fractions was delivered over 7 weeks (5 days per week) using intensity-modulated radiotherapy. The PSA showed a continuous downward trend (Fig. 4).

In August 2019, the patient reviewed with tPSA and fPSA was 1.88 ng/ml and 0.69 ng/ml, respectively. Chest CT showed both lungs were the same as before (Fig. 5B). While, bone scan indicated multiple bone metastases, which were given bisphosphonates regularly to prevent bone adverse events. The reexamination result of tPSA was 0.6 ng/ml and fPSA was 0.27 after 4 months. Imaging of bone metastasis was better than before.

The patient developed a low-grade fever (maximum temperature of 37.5 °C) without a clear inducement in the afternoon, accompanied by chest tightness, cough, and expectoration. Thereafter, Chest CT showed bilateral pleural effusion with atelectasis on February 25, 2020 (Fig. 5C). Further laboratory investigations were suggestive of positive mycobacterium tuberculosis complex DNA in pleural effusion and sputum, and ESR was significantly increased (101 mm/h). After the MDT discussion, the patient was diagnosed with a relapse of old PTB. He was treated with quadruple drugs (rifampicin + isoniazid + ethambutol + pyrazinamide) and abiraterone (1000 mg, qd) plus prednisone (it was reduced from 5 mg twice daily to 5 mg once daily.) according to MDT. Two months later, pleural effusion and atelectasis were relieved, and PSA was remained stable at a low level (Fig. 5D, Fig. 4). The patient achieved complete remission. Unfortunately, the follow-up revealed that the patient died in an accident in December 2020.

## 4 Discussion

Androgen deprivation treatment is the main treatment for metastatic prostate cancer, while combined androgen blockade therapy can prolong the overall survival and progression-free survival of prostate cancer patients.^[3]^ However, after 18 to 24 months of androgen deprivation treatment, prostate cancer cells can adapt to the low testosterone status by increasing the expression of their androgen receptor, can also generate testosterone in the tumor microenvironment by autocrine and paracrine, and activation of other signal pathways, which can remove the maximum inhibition of AR. Therefore, prostate cancer cells can still survive at castrated testosterone level and develop into castration-resistant prostate cancer.^[4]^ In our case, after 12 months of combined androgen blockade treatment, the PSA level of this patient was increased significantly and developed into metastatic castration-resistant prostate cancer (mCRPC), and the progression time was short and the development speed was fast, which may be related to the patient's higher Gleason score. The lowest baseline level of PSA was 0.37ng/ml, and the maintenance time was short, which may indicate that the patient progress to castration-resistant prostate cancer earlier. A large number of studies have also confirmed that the Gleason score is an independent predictor of prostate cancer recurrence, and the patients with a higher pathological grade have a higher risk of recurrence.^[5,6]^

Clinical studies have found that PSA may continue to rise in the early stage of docetaxel-based chemotherapy for some prostate cancer patients, but the clinical symptoms and imaging manifestations show a stable or improved trend, thereafter PSA shows a downward trend in the subsequent treatment, which is called “PSA flare”.^[7]^ However, after 5 chemotherapy courses, the PSA was still rising rapidly. Therefore, the possibility of “PSA flare” was ruled out. As such, prostate radiotherapy combined with abiraterone plus prednisone was performed following MDT, due to the higher Gleason score, the rapid development of PSA, and the larger tumor load. National Comprehensive Cancer Network and European Association of Urology guidelines also recommended that the patients with extracapsular invasion and seminal vesicle invasion should receive radiotherapy or early salvage radiotherapy.^[8]^ Large-scale clinical studies have also confirmed that regardless of whether mCRPC patients have received docetaxel chemotherapy in the past, abiraterone can still be the first choice of mCRPC, which can significantly prolong the overall survival rate.^[9]^

The patient's PSA showed a continuous downward trend after prostate radiotherapy combined with abiraterone plus prednisone. However, some studies have shown that there may also be a temporary increase of PSA in the early stage of abiraterone treatment, that is “PSA flare.”^[10]^ There was no increase of PSA in this patient after the therapy of abiraterone combined with prednisone, which may be related to the reduction of tumor load by concurrent radiotherapy. Robert et al^[11]^ considered that the increased PSA level, before radiotherapy, may be a prognostic indicator of radiotherapy and antiandrogen therapy for prostate cancer, and the antiandrogen treatment of patients receiving radiotherapy is related to the improvement of prognosis. Therefore, the PSA did not increase significantly and remained at a low level for a long time in the anti-androgen stage after radiotherapy. Satkunasivam et al^[12]^ found that radical surgery or intensity-modulated radiation therapy can significantly reduce the mortality of patients with metastasis than endocrine therapy alone. After 5 months of radiotherapy, a bone scan indicated multiple bone metastases, which was considered as ‘bone flare,” due to the patient's PSA was well controlled and showed a continuous downward trend. Cook et al^[13]^ studied bone scan changes of patients with metastatic prostate cancer undergoing endocrine therapy and considered that the occurrence of the “bone flare” phenomenon can improve the specificity and sensitivity of prostate cancer to hormone therapy. However, it is necessary to judge whether the patient is “bone flare” or “bone metastasis” in the treatment of mCRPC patients. If the general condition of the patients is improved and PSA is well controlled, the possibility of “bone flare” is high, otherwise, tumor progression cannot be excluded. Generally, the “bone flare” phenomenon is usually relieved after 6 months of treatment.^[14]^

The patient had a history of old PTB, and no obvious imaging changes were found during repeated reexamination, while tuberculosis has recurred after one year of continuous anti-tumor treatment, which was probably related to the long-term application of prednisone, and could also be related to the patient's weakened immunity caused by anti-tumor treatment for a long time. Studies have found that the eradication of mycobacterium tuberculosis requires alveolar macrophages and other phagocytes. If the human body's innate immunity cannot eliminate pathogens, active PTB can form localized infection through granuloma, which is mainly mediated by T cells. Tuberculous granuloma is composed of macrophages and a layer of peripheral lymphocytes, which play a role of protection to the host. Besides, the granuloma is the long-term survival nest of some tuberculosis bacilli in the body, which is the infection stage of latent tuberculosis, and any factor leading to immunosuppression may destroy the delicate balance of latent tuberculosis and cause tuberculosis infection to reactivate.^[15]^ The functions of glucocorticoids include but are not limited to: antagonize macrophages, inhibit macrophages to produce interleukin-1, interleukin-6, tumor necrosis factor, pro-inflammatory prostaglandins, leukotrienes, and inhibit the activity of activated antitumor and microbial macrophages, moreover, if the dosage of prednisone is increased by 1 g, the risk of tuberculosis will be increased by 23%.^[16]^

After the recurrence of tuberculosis, MDT discussed a balance point in the synchronous treatment of anti-tuberculosis and anti-tumor. Finally, prednisone was reduced to a dose of 5 mg every day. The PSA and tuberculosis were effectively controlled by synchronous endocrine and anti-tuberculosis treatment. Studies have confirmed that the combination of abiraterone and prednisone (5 mg/d) can be used for the treatment of high-risk metastatic castration-sensitive prostate cancer.^[17,18]^ However, in our case, the dosage of prednisone was reduced and the efficacy was still satisfactory, due to the recurrence of PTB. Therefore, further prospective, large sample, multicenter randomized controlled study is needed to determine whether prednisone can be reduced and whether prednisone (5 mg/d) plus abiraterone can also be applied to mCRPC patients as well as achieve a satisfactory effect.

## 5 Conclusion

In this case, we cannot, with complete certainty, say that this patient, or any patient, developed PTB due to the use of prednisone. Based on the current evidence, endocrine therapy is the basis, and local radiotherapy can help to reduce tumor load for advanced prostate cancer patients with high risk and high tumor burden. Early application of abiraterone is conducive to prolong the survival of patients. Prednisone can be appropriately reduced in patients with advanced prostate cancer complicated with old PTB, which can also achieve a satisfactory effect and alleviating the adverse reactions at the same time. More prospective trials are warranted before a definite recommendation could be drawn.

## Acknowledgments

The authors thank the patient for consenting to the publication of this case report.

## Author contributions

**Data curation:** Xiaojuan Zhu, Zhenguo Shi, Shegan Gao, Xiaohong Wang, Pei Wang, Chongzhao Kang, Fangzong Zhao, Peng Hou.

**Formal analysis:** Xiaojuan Zhu, Zhenguo Shi, Chongzhao Kang, Peng Hou.

**Investigation:** Shegan Gao, Xiaohong Wang, Chongzhao Kang, Fangzong Zhao.

**Methodology:** Xiaojuan Zhu, Zhenguo Shi, Pei Wang.

**Resources:** Xiaojuan Zhu, Zhenguo Shi, Chongzhao Kang, Fangzong Zhao.

**Supervision:** Xiaojuan Zhu, Zhenguo Shi, Shegan Gao.

**Writing – original draft:** Xiaojuan Zhu.

**Writing – review & editing:** Xiaojuan Zhu, Zhenguo Shi.
